# Implementation of Physical Activity in US Elementary Schools: The Role of Administrative Support, Financial Resources, and Champions

**DOI:** 10.3390/ijerph18094476

**Published:** 2021-04-23

**Authors:** Blake Densley, Hannah G. Calvert, Peter Boedeker, Lindsey Turner

**Affiliations:** 1 Center for School and Community Partnerships, College of Education, Boise State University, 1910 University Drive, Boise, ID 83725-1742, USA; blakedensley@u.boisestate.edu (B.D.); hannahcalvert898@boisestate.edu (H.G.C.); 2 College of Education, Boise State University, Boise, ID 83725-1745, USA; peterboedeker@boisestate.edu

**Keywords:** physical activity, implementation, elementary school, facilitators, movement integration

## Abstract

The intentional integration of physical activity in elementary school classrooms—including brief instructional breaks for activity, or integration into lessons—can benefit children’s physical activity and education outcomes. Teachers are key implementation agents, but despite physical activity in the classroom being an evidence-informed practice, many teachers do not regularly implement it. The aim of this study was to obtain updated nationally representative prevalence estimates in United States public elementary schools, regarding four key outcomes: (1) school adoption of physically active lessons (PA lessons); (2) school adoption of physical-activity breaks (PA breaks); (3) penetration in the classroom, defined as ≥50% of teachers using PA breaks; and (4) dose, defined as an average of at least 50 min per week of PA breaks. We examined variations in outcomes by school demographic characteristics, and by three factors hypothesized to be implementation facilitators (administrative support, financial resources, and presence of a wellness champion at the school). In the 2019–20 school year, surveys were distributed to a nationally representative sample of 1010 public elementary schools in the US; responses were obtained from 559 (55.3%). The weighted prevalence of schools reporting adoption of PA lessons was 77.9% (95% CI = 73.9% to 81.9%), and adoption of PA breaks was nearly universal at 91.2% (95% CI = 88.4% to 94.1%). Few demographic differences emerged, although adoption of PA lessons was less prevalent at higher-poverty schools (73.9%) and medium-poverty schools (77.0%) as compared to schools with lower poverty levels (87.1%; *p* < 0.01). Across all four outcomes, associations emerged with facilitators in multivariable logistic regression models. The prevalence of adoption of PA lessons, adoption of PA breaks, and dose of PA breaks were all significantly higher at schools where administrative encouragement occurred more frequently. Financial support was associated with implementation outcomes, including adoption of PA lessons, and penetration and dose of PA breaks. Presence of a champion was associated with higher prevalence of reporting adoption of PA lessons. School leaders can play a crucial role in supporting teachers’ implementation of PA breaks and lessons in the classroom, through providing financial resources, encouragement, and supporting champions. Effective school-leadership practices have the potential to positively impact students at a large-scale population level by supporting implementation of PA lessons and breaks.

## 1. Introduction

Despite overwhelming evidence about the benefits of regular physical activity [1], most children worldwide do not meet guidelines [2]. Schools are a crucial setting for ensuring adequate opportunities for physical activity, and health-promoting school environments can benefit many students [3,4]. One approach to increasing activity during the school day—particularly at the elementary school level—is through “movement integration”, which has been defined as “infusing physical activity, at any level of intensity, within general education classrooms during normal classroom time” [5]. Movement integration can take several forms, including short breaks from instruction where the entire class is physically active for a brief period, typically called physical-activity breaks (PA breaks), or through physically active lessons (PA lessons), which integrate academic content with intentional movement [6]. An example of a PA lesson in mathematics might include having students walk in place or skip while reciting times tables [7]. The United States (US) Department of Health and Human Services [8] finds sufficient evidence that classroom-based movement integration is effective for increasing physical activity among children and youth, and several recent systematic reviews document the benefits of PA breaks and PA lessons for increasing students’ physical-activity levels [9,10,11]. A systematic review and meta-analysis of 42 studies of PA lessons found significant increases in overall physical activity levels (*d* = 0.32), instructional-time physical activity (*d* = 2.33), instructional-time educational outcomes (*d* = 0.81), and overall educational outcomes (*d* = 0.36) [10]. A second systematic review and meta-analysis of 22 studies of PA breaks noted that most found significant effects for physical-activity outcomes, with a significant meta-analysis effect for increased step counts; in addition, time on task was increased by PA breaks [9]. A third systematic review of 18 studies also found that PA lessons and breaks increased physical-activity outcomes [11]; in addition, the review concluded that time on task was improved after a dose of at least 10 min of moderate-to-vigorous activity, or 5 min of vigorous activity, suggesting that dose is important for achieving significant benefits. Recent reviews have noted inconclusive results for the impact of PA breaks and lessons on weight or health outcomes, as few studies have yet assessed these outcomes [9,11].

Despite the growing evidence about the benefits of PA lessons and breaks, it remains unclear to what extent these practices occur in schools, and what factors are associated with implementation. Periodic assessment of school environments nationwide can provide crucial guidance about areas of progress and opportunities for improvement [1,8]. However, in the US [12], as in many countries [13,14,15,16], national surveillance options about school practices are relatively limited. Internationally, most surveillance projects have focused on physical education in schools, noting the need for physical activity throughout the school day, but not specific to PA lessons and breaks [13,14,16]. As part of Finland’s Schools on the Move program, which includes school participation from 75% of all primary schools, nearly half of all teachers surveyed reported the use of PA lessons, and around 60% broke up classroom instructional time with PA breaks [15]. Nationally representative data were collected in the US in 2014 by the Centers for Disease Control and Prevention, in which survey respondents at 43.3% of elementary schools reported that students “participate in regular PA breaks outside of physical education, during the school day” [17]. This does not provide an indication of how many teachers at each school use PA breaks, but such estimates were reported by Turner and Chaloupka [18] using a nationally representative survey of principals at 640 public US elementary schools, also in the 2013–2014 school year. PA lessons were reported to be adopted at 71.7% of schools, and PA breaks at 75.6% of schools. At schools where PA breaks were reported to be adopted, principals estimated that 45.6% of teachers, on average, were using PA breaks, and most principals estimated that students received <10 min of activity per day, on average. In other words, prior surveillance suggests that there is reasonable school-level adoption of movement-integration practices in US elementary schools, but within-school use of these practices was much lower, and daily dose was typically below recommendations. Several implementation outcomes [19] are relevant to improving the understanding of movement integration, including adoption (i.e., the use of an evidence-based practice), as well as organizational penetration to subsystems (i.e., classrooms), and dose (i.e., the extent of movement-integration opportunities available to students). While other studies have explored the extent to which teachers within a school adopt movement integration [20,21,22,23,24,25], there is a need for estimates of organizational penetration rates and dose in a national sample of schools.

Furthermore, in addition to the need for national surveillance of various implementation outcomes, there is a need to explore the prevalence of factors that often support or hinder school-level and classroom-level implementation. In a recent systematic review of barriers and facilitators of movement integration, Michael and colleagues identified four main barriers, including lack of time, resources, space, and administrative support [26]. These are consistent with barriers and facilitators noted in a systematic review of the determinants of implementation of broader school physical-activity policy initiatives (including PA breaks and lessons and other practices), among which environmental context/resources and social influences/support were key determinants [27]. The lack of resources is a common barrier to implementation of any new initiative, and movement-integration-specific resource barriers involve lack of equipment, materials, and curricula, all of which require funding [28,29]. Lack of administrative support hinders implementation of movement-integration practices [30,31,32,33], and an expert review of factors related to implementation of physical-activity interventions concluded that administrative support was a key predictor of success [34]. While administrators or organizational leaders are important, other agents can also serve as implementation champions in schools. Much work explores the roles and characteristics of physical-activity leaders or champions, considered by many to be crucial for successful implementation of initiatives [35,36,37]. A physical-activity champion can be a leader who trains staff on movement integration, provides encouragement, and facilitates opportunities for staff to participate in physical activity [38]. While many interventions to increase school-based physical activity recognize the role of champions, few nonintervention studies have assessed whether schools organically have such champions in place, and whether having a champion is associated with differences in practices. More research is necessary to explore the prevalence of school characteristics that may facilitate movement integration, including financial resources, administrator support, and the prevalence of champions.

The aim of this manuscript is to present the results of a nationally representative survey of US public elementary schools, conducted to examine the prevalence of movement-integration implementation outcomes and hypothesized facilitators in 2019–2020. Outcomes were compared with estimates previously reported about US practices in 2013–2014. In addition, variations in implementation outcomes were considered based on school demographic characteristics and the three hypothesized implementation facilitators.

## 2. Materials and Methods

This study utilized cross-sectional data from a survey of elementary schools in the US, conducted during the 2019–2020 school year. This project used the same sample frame as a prior survey conducted by the current authors in 2013–2014 and described in detail elsewhere [18,39]. The sample frame and analytic weights were developed by survey sampling methodologists at the Institute for Social Research at the University of Michigan. The sample was initially selected through stratified simple random sampling, designed to be nationally representative of US public elementary schools (i.e., those containing a grade 3 class). Due to differing grade-level arrangements for schools across the country (i.e., kindergarten to grade 4; grades 1–5, etc.), those serving at least one grade 3 class (students typically 9 years of age) were considered to be elementary schools for this study. A two-stage selection approach was used, first developing a nationally representative sample of districts based on state location, urbanicity, and number of students. Thereafter, schools were selected within each district group, with the measure of size for selection of schools based on the number of grade 3 students. Additional details are available elsewhere [39].

### 2.1. Data Collection Overview

Starting with the sample of 1045 elementary schools from 2013–2014, prescreening was conducted to verify mailing addresses and obtain e-mail addresses. Of the original 1045 schools, 1010 were determined to still be eligible (i.e., open, operating, and an elementary school). Fielding of the survey began on 26 August 2019 with a mailed survey and invitation letter to school principals. Respondents had the option to either fill in the paper survey, or complete the survey electronically through Qualtrics. Reminders were delivered by e-mail, telephone calls, and a second mailing of the survey. Recruitment closed on 28 February 2020, with responses from 559 schools (55.3% response rate). The primary respondent of interest was the principal, but delegation was allowed to other respondents as appropriate, including assistant principals, teachers, or others with knowledge of school practices. Respondents were offered a $50 online gift card. The Institutional Review Board at Boise State University approved the study protocol (101-SB19-151).

### 2.2. Measures

The full survey included 66 items, covering topics including: school nutrition; physical education and physical activity; and wellness-policy implementation. Although the surveys were pretested during initial development [39], the items used in these analyses were not extensively validated. The current analyses used seven items from the survey.

#### 2.2.1. Measures: Implementation Outcomes

***Adoption of PA lessons*** was assessed with one item: “Do any classroom teachers at your school provide active learning opportunities by incorporating physical activity into existing lessons (e.g., having children spell words by jumping on a mat with letters, counting while doing jumping jacks, etc.)”, with response options of “yes”, “no”, and “don’t know”.

***Adoption of PA breaks*** was assessed with the item: “Some classroom teachers offer brief breaks during the school day (other than PE and recess time) for movement or brief bursts of physical activity in the classroom (e.g., Take10!, Energizers). Do any teachers at your school provide such activity breaks?” Response options were “yes”, “no”, and “don’t know”. If the respondent answered in the affirmative to this item, two follow-up items were used to assess penetration and dose.

***Penetration of PA breaks*** was assessed with the open-ended item: “How many teachers at the school use activity breaks?” A penetration percentage was calculated by dividing the survey respondents’ report of how many teachers use PA breaks by the number of total full-time equivalent teachers, as reported in the school demographic files from the Common Core of Data [40].

***Dose of PA breaks*** was assessed with the open-ended item: “How many minutes per week are third-grade students active in activity breaks, not including physical education and recess?”

#### 2.2.2. Measures: Implementation Facilitators

Three items were used to assess key school-level implementation facilitators.

***Administrator support for PA breaks*** was addressed with the item: “Does the school administrator/principal provide encouragement to classroom teachers to use physical activity breaks?”, with response options of “never”, “rarely”, “sometimes”, and “often”.

***Financial support for PA breaks*** was assessed with the item: “Is financial support (from the district or the school) available to teachers who want to purchase curricula/resources for physical activity in the classroom?” Response options were “yes”, “no”, and “don’t know”.

***Presence of a school wellness champion*** was measured with an item using a definition of a champion provided elsewhere [41,42]: “Is there one or more persons at your school who could be considered a champion for wellness? A champion can be defined as someone who is ‘dedicated to supporting wellness and overcoming indifference or resistance from others…someone who is willing to risk informal status and reputation because they believe so strongly in student wellness.’ ” Response options were “yes”, “no”, and “don’t know”.

#### 2.2.3. Measures: Contextual Covariates

The sampling frame included the National Center for Education Statistics unique identification number for each school, which was used to merge in demographic information. Demographic characteristics were acquired from the Common Core of Data [40] and were used for calibration of analytic weights, as sample descriptors, and as covariates in statistical models. Variables included school region, school locale, school size, and student racial/ethnic composition and socioeconomic status. US census region was classified based on each school’s location in a state (Northeast, Midwest, South, or West). Locale was classified as urban, suburban, town, or rural. The total number of students was used as an indicator of school size. For consistency with our previous analyses in 2013–2014 [18], the same coding strategy was used for demographic covariates. Size included three groups based on number of students (≤450, 451 to 621, and >621). Each school’s student racial/ethnic composition was coded into one of four exhaustive and exclusive categories: predominantly (≥66%) White non-Latino, majority (≥50%) Black non-Latino, majority (≥50%) Latino, and other (diverse, or majority Asian or Native American). The percentage of students eligible for free/reduced-priced meals was used as a proxy for socioeconomic status at the school level. Students with household incomes of up to 185% of the US federal poverty level are eligible for free/reduced-priced meals, and the overall percentage of eligibility is a common metric for school socioeconomic composition in the US [43]. For consistency with our previous analyses in 2013–2014, the same strategy was used [18], coding schools as lower socioeconomic status (>66% eligible), moderate (>33% to ≤66% eligible), and higher (≤33% eligible).

### 2.3. Statistical Analysis

#### 2.3.1. Analysis Overview

Analyses were completed using STATA 16 (StataCorp LP, College Station, TX, USA). The svyset and svy commands were used to account for the survey design, including clustering within district, sampling stratification, and weighting. The default method of variance estimation (Taylor linearization) was used, and single units were treated as certainty estimates rather than missing values; additional commands (e.g., bootstrapping, postestimation) were not used [44]. Sampling weights were included with the original sampling frame, developed to allow for national inferences. These weights accounted for the probability of selection and school size (number of grade 3 students); more details are available elsewhere [39]. Postresponse calibrations were made to the weights, to account for propensity to respond in 2019–2020, using school demographic characteristics associated with response status (yes/no).

#### 2.3.2. Preliminary Analyses and Data Recoding

Data were first explored with frequencies and weighted percentages for all predictor, contextual covariate, and outcome variables. The proportion of missing data for each survey item and demographic characteristics was minimal, and was highest for items assessing number of teachers using PA breaks (4.7%) and PA break minutes/dose (7.6%). Chi-square tests compared schools that had valid data on this item (*n* = 449) versus those with missing data (*n* = 43); no significant demographic differences were noted, therefore listwise deletion was used for analyses. After initial examination of response patterns showing relatively few “don’t know” responses, those responses were combined with “no” responses (coded = 0) for adoption of PA lessons and breaks.

For penetration of PA breaks (percentage of teachers adopting breaks), the distribution was flat and not normal (M = 0.53, SD = 0.30, kurtosis = −1.13), therefore proportions were recoded into four groups (>0% to <25% of classrooms; ≥25% to <50%; ≥50% to <75%; and ≥75%). Schools that reported no adoption of PA breaks were coded as 0% penetration.

Dose of PA breaks was based on reported average number of minutes of breaks per week; the distribution was skewed and kurtotic (M = 32.21 min, SD = 29.30, skew = 3.24, kurtosis = 19.79). This variable was converted to three categories representing low (≤25 min/week), medium (>25 to <50 min/week), and high (≥50 min/week) levels of engagement. These categories correspond to an average of less than 5 min per day for the low group, to greater than 10 min per day for the high group, and were selected based on recommended doses of physical activity in the scientific literature, whereby at least 10 min of movement integration per day is widely considered a meaningful dose [45,46,47]. Schools that reported no adoption of PA breaks were coded as dose of zero minutes.

#### 2.3.3. Analyses

To examine whether changes occurred in the reported prevalence of adoption of PA breaks and lessons between 2013–2014 and 2019–2020, weighted prevalences with 95% confidence intervals (CIs) were computed. Estimates from 2019–2020 were compared against data gathered in 2013–2014 using the same sampling frame [39]. Nonoverlapping CIs were assumed to indicate statistically reliable differences over time.

Four separate multivariable logistic regression models were conducted to examine each of the movement integration outcomes. Adoption of PA lessons and PA breaks were coded as yes/no; however, additional recoding was used for analyses predicting penetration and dose, based on meaningful empirical cutpoints. For PA break penetration, a logistic regression model tested penetration rates of <50% versus ≥50%. This decision was based on prior work showing a mean of 45.6% of teachers using PA breaks [18], and other work showing that 50% of teachers were regularly scheduling physically active breaks in their classroom [48]. Therefore, a simple majority of teachers was considered for penetration. For dose of PA breaks, a threshold of ≥50 min/week was compared versus lower dose, based on 10 min per day (i.e., 50 min per week), being widely considered a meaningful dose in the previously published literature [45,46,47].

For each model, all school demographic characteristics were included as contextual covariates, and the three hypothesized implementation facilitators were entered simultaneously. A series of contrasts were used for entry of each predictor variable into the regression model, comparing each level with the referent group for that construct (e.g., comparing medium-sized schools with smaller schools, and larger schools with smaller schools). Adjusted prevalences were calculated, which indicated the percentage of schools engaging in each practice, at each level of the predictor, while accounting for all other variables in the model.

## 3. Results

### 3.1. School Demographic Characteristics

Responding schools represented a diverse range of demographic characteristics (see Table 1), from across all regions of the US, and were diverse in size, locale, and student socioeconomic and racial/ethnic composition. Among the 559 respondents, 273 (48.8%) were principals and 286 (51.2%) were in other roles.

### 3.2. Survey Results

Table 2 presents descriptive statistics for implementation outcomes and facilitators. Respondents at 77.9% of schools reported the adoption of PA lessons, while 91.2% reported adoption of PA breaks. Only 21.5% of schools reported a dose of PA breaks of at least 50 min per week. Few respondents indicated that administrative support occurred “never” (2.9%), so this response was combined with the “rarely” group for further analyses.

### 3.3. Changes in Prevalence of Reported Adoption of PA Lessons and PA Breaks

In 2019–2020, the weighted prevalence of adoption of PA lessons was 77.9% (95% CI = 73.9% to 81.9%). This did not represent a significant change in prevalences previously reported [18] from the 2013–2014 school year (71.7%, 95% CI = 67.9% to 75.5%). Reported adoption of PA breaks in 2019–2020 was nearly universal, with a weighted prevalence of 91.2% (95% CI = 88.4% to 94.1%), an increase of nearly 16 percentage points from 2013–2014 (75.6%, 95% CI = 71.7% to 79.4%).

### 3.4. Characteristics Associated with Each Outcome

Model 1 (Table 3) revealed that, in a multivariable logistic regression framework, adoption of PA lessons varied significantly by several predictors. Adoption of PA lessons was reported by more schools in the South (85.4% of schools) than the Northeast (71.1%, *p* = 0.024); it was also reported more frequently at higher socioeconomic status schools (87.1%) as compared to middle socioeconomic status schools (77.0%, *p* = 0.036) and compared to lower socioeconomic status schools (74.0%, *p* = 0.024). In addition, adoption of PA lessons varied by implementation facilitators. Adoption of PA lessons was more common where a school champion was present (82.2%) versus not present (71.8%, *p* = 0.014). It was also more common in schools where administrator support happened often (84.9%) than when support was reported to occur never or rarely (57.6%; *p* < 0.001). Adoption of PA lessons was also reported more at schools that provided financial support (83.2%) than where financial support was not provided (73.7%; *p* = 0.011).

With regard to adoption of PA breaks (Model 2; Table 3), several school characteristics were associated with the prevalence of this outcome, which was higher in the Midwest (94.9%, *p* = 0.01) and South (93.6%, *p* = 0.02) compared to the Northeast (82.6%). At schools where the administrator provided encouragement often, 97.8% had adopted PA breaks, versus 78.2% of schools where encouragement happened never or rarely (*p* < 0.001).

Model 3 (Table 4) examined predictors of classroom penetration (≥50% of teachers). The only implementation facilitator associated with this outcome was whether schools had financial support: those with a budget for PA breaks were more likely to report a penetration rate of at least 50%, as compared to schools that did not receive support (58.1% versus 43.8%, *p* < 0.01).

Model 4 (Table 4) examined which predictors were associated with schools reporting that a dose of ≥50 min of PA breaks were provided per week. This dose was less common among schools in the West (adjusted prevalence = 9.9%), as compared to schools in the Northeast (34.7%, *p* < 0.01). Administrative support was significantly associated with this outcome, whereby a dose of ≥50 min of PA breaks per week was more common at schools that received administrative support often (27.9% of schools) compared to those that never or rarely received support (4.9%, *p* < 0.01). Similarly, a higher dose was reported at schools that had financial support (27.0%) than those that did not (15.9%, *p* < 0.01). The presence of a wellness champion was not significantly associated with dose.

## 4. Discussion

This study provides nationally representative estimates of movement integration adoption, penetration, and dose in US elementary schools in 2019–2020. A key finding was that adoption of PA breaks was nearly universally reported, with a weighted prevalence of 91.5%. This was a significant increase of nearly 16 percentage points from the estimate of 75.6% found in 2013–2014 [18]. The demographic characteristics of this survey sample were similar to those in the dataset for the prior report, and both were obtained using the same sampling frame [18], so the comparison suggests that there may have been a reliable increase in prevalence of PA breaks across the US over the past seven years.

Although there is more evidence about the educational benefits of PA lessons—that is, the intentional combination of movement with academic lessons—particularly for mathematics outcomes [49], adoption of PA lessons was reported less frequently than PA breaks. In 2019–2020, the weighted prevalence of PA lessons was 77.9%, occurring in about 3 in 4 US elementary schools, and not appearing to significantly change from 71.7% in the 2013–2014 school year. A distinguishing characteristic between PA breaks and lessons is the planning necessary to integrate activity into an academic lesson. Time for planning has been identified by teachers as a persistent barrier to movement integration, along with a lack of financial resources to purchase curricular resources [26], which may explain the lack of significant increases over time in reported adoption of PA lessons. However, given recent experimental results demonstrating that, in a head-to-head comparison trial, PA lessons improved mathematics outcomes more than PA breaks or a no-activity control condition [49], it is important to understand more about barriers to the use of lessons that integrate activity with academic content, and to focus efforts on improving implementation of PA lessons. Both PA lessons and PA breaks appear to significantly increase students’ physical-activity levels [11], so more work is needed to explore mechanisms through which differential impacts on education outcomes might occur.

Another goal of the current work was to examine whether demographic disparities that had been noted among elementary schools in 2013–2014 were still evident in 2019–2020. In 2013–2014, adoption of PA lessons was reported less commonly in schools serving predominantly Latino students, as compared to schools serving predominantly white students [18]. Furthermore, adoption of PA breaks was reported less commonly in schools in higher-poverty communities, compared to the lowest-poverty schools [18]. The current data show few demographic disparities in implementation outcomes in 2019–2020, as reported adoption of PA lessons or PA breaks did not vary at all by school racial/ethnic composition; however, there was still a disparity in adoption of PA lessons based on school socioeconomic characteristics, whereby they were less prevalent at the highest-poverty schools (73.9%) and medium-poverty schools (77.0%), as compared to schools with the lowest poverty levels (87.1%). This is perhaps not surprising, given the many challenges faced by schools that serve students from higher-poverty communities, where many risk factors are present, and school budgets must be allocated to the most pressing needs. Nevertheless, this is an important area for future work, as addressing movement integration implementation among low-resource schools will be crucial for improving health equity at a population level [50].

While the reported adoption of PA breaks in schools across the US increased over time, this metric says little about the frequency or duration of their use, nor penetration, in terms of the percentage of classrooms in which PA breaks are estimated to be regularly used. Classroom penetration of ≥50% of teachers was modest, occurring at 49.9% of schools in 2019–2020. Dose was also modest, with 40.3% of schools reporting that teachers used PA breaks for, on average, at least 25 min per week, and 21.5% of schools reporting that teachers used PA breaks for at least 50 min per week. Importantly, despite the substantial increase in the prevalence of reported adoption of PA breaks at the school level, there is still much room for growth in classroom-level penetration, as these estimates show that for half of elementary schools, it is still not the norm for teachers to use PA breaks. In addition, a racial/ethnic disparity was noted in penetration, with schools serving majority Latino students being less likely to report penetration rates of ≥50% of classrooms. This illuminates an important focus for future efforts to improve equitable opportunities for all students, by considering which implementation support strategies may improve adoption at the classroom/teacher level. For example, more work is needed to explore factors associated with the adoption of movement-integration practices by teachers, with a specific focus on teachers at schools serving high proportions of students of color or those living with household poverty. It is likely that these teachers face many competing demands [33,50], but it also remains important to identify strategies to improve the implementation of health- and academic-promoting practices at these schools.

School-level reports of adoption of PA breaks and lessons were both significantly higher at schools where administrative encouragement occurred more frequently. Notably, in the case of adoption of PA breaks, which was reported by a majority of schools, the potential to reach almost full adoption appears possible (97.8%) with the presence of a leader who supports its use. While the penetration of PA breaks was not significantly associated with leadership support, higher dose was estimated at schools where administrators often provided encouragement. The association between leadership from school administrators and these outcomes is consistent with results of a systematic review by Michael and colleagues [26], which found that effective leadership was one of the crucial institutional-level facilitators of successful implementation of movement-integration practices. We also found that financial support was associated with more adoption of PA lessons; however, adoption of PA breaks did not differ, but that may be because of a ceiling effect where adoption was nearly universal. Interestingly, financial support was associated with two of the more-novel implementation outcomes explored in these analyses, both classroom-level penetration and dose. These results highlight the importance of having tangible resources available to support the implementation of PA breaks and lessons by teachers who wish to do so. Some curricula such as Energizers [51] are freely available, and the US Centers for Disease Control and Prevention has a free implementation support guide [6]. Online services such as GoNoodle (www.gonoodle.com, accessed on 22 April 2021) offer free access to many resources, with upgrades via fee-based subscription. District and school administrators can support movement integration by providing relatively modest amounts of funding to facilitate access to curricula and other resources.

Reported adoption of PA lessons was higher at schools with a wellness champion (82.2%) than those without (71.8%), but champions were not associated with PA break adoption, penetration, or dose. Previous and ongoing intervention trials have explored whether empowering champions can improve physical-activity practices in schools [52,53], however, the current work is unique in examining whether champions already exist in US elementary schools. Nearly half of respondents (56.9%) noted that such a champion is present at their school; while this is encouraging, there remains much room for improvement. It is also important to note that the survey item asked broadly about wellness champions, some of whom may instead focus on nutrition or other aspects of wellness not specifically including physical activity, thus we believe there is even more need for broadly empowering physical activity champions at schools.

As with any study, these results are subject to several limitations. Social desirability is always a concern with surveys, and respondents may also have had inaccurate or incomplete knowledge of the practices at their school. While the survey items used in this study have face validity, the lack of formal development and testing of validity of survey-reported items is a limitation. In this study, as with others, adoption at a school level could reflect that as few as one teacher in the entire school is a regular user of PA breaks or lessons; therefore, this study also explored additional implementation outcomes, specifically considering penetration to the classroom level and dose of PA. However, it is unclear to what extent school-level respondents are able to provide valid or reliable estimates of in-classroom practices, which particularly impacts our estimates of penetration and dose. The school principal was the intended recipient of our survey, and about half of respondents were principals (48.8%). Principals are typically aware of their teachers’ instructional practices, thus it is likely that the principal could estimate which teachers used movement integration, so estimates of penetration may be reasonable. However, it is likely to be more difficult for principals or other respondents to accurately estimate dose (minutes of PA breaks). In addition, respondents were asked to report on principal encouragement, and where the respondent was the principal, this construct may have been impacted by social desirability, with principals over-reporting support, or inaccurately estimating how much support is perceived by classroom teachers. Exploring teacher perspectives on movement integration is necessary to better understand how implementer characteristics (e.g., knowledge, skill, confidence, attitudes) are associated with classroom-level adoption/penetration, fidelity, dose, and other implementation outcomes. Other studies have used teacher self-reports to explore adoption, fidelity, dose, and other outcomes, with prior approaches including surveys [32,54] and daily or weekly tracking logs [33,49,55]; both approaches would allow for more detailed and reliable classroom-level outcomes than estimation by a school-level reporter. Observational studies would reduce some of the response biases associated with survey self-reporting or tracking logs, but are logistically and financially burdensome and not feasible for national surveillance purposes. The statistical approach is also subject to several limitations, primarily due to the collapsing of penetration and dose into two groups due to the distribution of these variables; although this allowed for comparison of the prevalence of a “higher” penetration rate (relative to a lower group), and a “higher” dose of PA breaks, other cutpoints could have been used. We established categories based on meaningful criteria in the literature, but we acknowledge that dichotomizing can obscure important information and is not ideal, and that other coding schemes may be preferable. Lastly, this study only explored cross-sectional associations between a few hypothesized implementation facilitators and movement-integration outcomes; there are likely many other important determinants of implementation outcomes that should be explored. Causality cannot be assessed cross-sectionally, and because this was not an intervention trial, it is unclear whether changes in facilitators may change implementation outcomes. Randomized controlled trials are needed to examine the effectiveness of strategies to modify implementation facilitators such as financial and administrative support.

## 5. Conclusions

Evidence continues to accumulate about the value of providing multiple opportunities for students to engage in physical activity during the school day, as part of a comprehensive school approach [4,56]. In this large, nationally representative sample of US public elementary schools, we found nearly universal adoption of PA breaks, but room for improvement in PA lessons, as well as penetration and dose of PA breaks at the classroom level. School-level facilitating factors (administrator support, financial resources, and the presence of school champions) help to explain variations in implementation outcomes. Administrators play a crucial role in supporting teachers’ use of these practices within the classroom, including provision of financial resources, encouragement, and empowering champions who advocate for movement integration. Little research has studied the perceptions of school administrators about movement integration, representing an important area for additional work. Effective school leadership to facilitate teachers’ implementation of movement-integration practices has the potential to positively impact physical activity, behavior, and academic outcomes for children at a population level.

## Figures and Tables

**Table 1:** Demographic characteristics of 559 participating US public elementary schools.

Demographic Characteristic	N (Unweighted)	% (Weighted)
School size		
Small (≤450)	276	49.6
Medium (>450 to 621 students)	171	29.1
Large (>621 students)	111	21.3
Socioeconomic status (% students eligible for FRPL ^1^)		
Higher (≤33% eligible)	139	21.5
Middle (>33% to ≤66% eligible)	200	33.9
Lower (>66% eligible)	192	40.7
Locale		
Urban	114	31.5
Suburban	205	37.5
Township	79	9.9
Rural	161	21.2
US census region		
Northeast	107	15.6
Midwest	165	23.8
South	196	34.6
West	91	26.1
Race/ethnicity of enrolled students		
Predominantly (≥66%) White non-Latino students	249	34.2
Majority (≥50%) Black non-Latino students	43	8.6
Majority (≥50%) Latino students	86	21.6
Other majority or diverse student composition	181	35.6

^1^ Note: FRPL = free/reduced-priced lunch (a proxy for financial disadvantage).

**Table 2:** Descriptive statistics for survey responses and key variables of interest.

Survey Variable	N (Unweighted)	% (Weighted)
Use of PA lessons		
Yes	446	77.9
No	37	6.0
Don’t know	70	16.1
Use of PA breaks		
Yes	511	91.2
No	9	1.6
Don’t know	33	7.1
Classroom penetration of PA breaks		
>0 to <25% of teachers (or not adopted at school)	135	26.6
≥25% to <50% of teachers	116	23.4
≥50% to <75% of teachers	121	22.1
≥75% of teachers	145	27.8
Dose of PA breaks per week		
Lower (≤25 min or not adopted at school)	291	59.7
Medium (>25 to <50 min)	99	18.8
Higher (≥50 min)	124	21.5
School administrative support		
Never	14	2.9
Rarely	31	5.6
Sometimes	215	39.2
Often	285	49.7
Financial support		
Yes	268	46.9
No	164	31.0
Don’t know	118	22.1
Presence of a school wellness champion		
Yes	307	60.1
No	114	20.3
Don’t know	102	19.6

**Table 3:** Summary of results of logistic regression models to predict implementation outcomes.

Model and Outcome Variable	Model 1 Physically Active Lessons			Model 2 Physical-Activity Breaks		
Predictor Variables	Odds Ratio	95% CI	Adjusted Prevalence	Odds Ratio	95% CI	Adjusted Prevalence
School size (# of students)						
Small (≤450)	1.00		76.1	1.00		92.9
Medium (>450 to 621)	1.13	0.60, 2.13	77.9	1.09	0.43, 2.77	93.4
Large (>621)	1.55	0.75, 3.21	82.2	0.59	0.23, 1.57	89.2
Socioeconomic status (% FRPL ^1^)						
Higher (≤33%)	1.00		87.1	1.00		93.9
Middle (>33% to ≤66%)	0.45 *	0.21, 0.95	77.0	0.68	0.19, 2.43	91.6
Lower (>66%)	0.37 *	0.155, 0.88	73.9	0.73	0.17, 3.18	92.1
Locale						
Urban	1.00		78.6	1.00		92.5
Suburban	0.87	0.43, 1.76	76.6	1.40	0.58, 3.36	94.4
Township	1.25	0.48, 0.26	81.6	0.69	0.19, 2.50	90.0
Rural	0.94	0.42, 2.09	77.6	0.57	0.15, 2.19	88.4
Region						
Northeast	1.00		71.1	1.00		82.6
Midwest	2.07	0.87, 4.92	82.4	4.74 *	1.45, 15.48	94.9
South	2.65 *	1.14, 6.13	85.4	3.68 *	1.19, 11.34	93.6
West	0.77	0.34, 1.73	66.2	2.80	0.88, 8.85	92.0
Student race/ethnicity						
Predominantly (≥66%) White	1.00		77.3	1.00		93.9
Majority (≥50%) Black	0.75	0.26, 2.16	72.8	0.61	0.10, 3.71	90.9
Majority (≥50%) Latino	1.63	0.64, 4.11	83.8	0.37	0.09, 1.53	86.7
Other/diverse	0.84	0.42, 1.67	74.7	1.10	0.36, 3.34	94.4
School administrative support						
Never/rarely	1.00		57.6	1.00		78.2
Sometimes	2.35	0.92, 5.97	74.2	2.42	0.79, 7.40	88.9
Often	4.95 ***	2.10, 11.68	84.9	14.40 ***	3.58, 57.88	97.8
Financial support						
No/Don’t know	1.00		73.7	1.00		91.4
Yes	1.93 *	1.16, 3.21	83.2	1.36	0.58, 3.18	93.3
School wellness champion						
No/Don’t know	1.00		71.8	1.00		90.1
Yes	1.98 *	1.15, 3.41	82.2	1.72	0.74, 3.99	93.7
Number of schools in model	488			489		

^1^ Note: FRPL = free/reduced-priced lunch (a proxy for financial disadvantage). * *p* < 0.05, *** *p* < 0.001.

**Table 4:** Summary of results of logistic regression models to predict implementation outcomes.

Model and Outcome Variable	Model 3 PA Breaks Penetration Rate ≥ 50%			Model 4 PA Breaks Dose ≥ 50 min/Week		
Predictor Variables	Odds Ratio	95% CI	Adjusted Prevalence	Odds Ratio	95% CI	Adjusted Prevalence
School size (# of students)						
Small (≤450)	1.00		49.0	1.00		23.2
Medium (>450 to 621)	1.49	0.90, 2.47	57.8	0.65	0.36, 1.20	17.2
Large (>621)	0.84	0.45, 1.55	45.1	0.95	0.42, 2.12	22.4
Socioeconomic status (% FRPL ^1^)						
Higher (≤33%)	1.00		51.9	1.00		27.5
Middle (>33% to ≤66%)	1.02	0.60, 1.78	52.5	0.81	0.40, 1.63	24.0
Lower (>66%)	0.86	0.46, 1.61	48.5	0.45	0.18, 1.14	16.0
Locale						
Urban	1.00		51.9	1.00		17.8
Suburban	1.19	0.70, 2.01	55.6	1.48	0.65, 3.36	23.3
Township	0.64	0.30, 1.35	42.0	1.49	0.52, 4.28	23.4
Rural	0.71	0.38, 1.35	44.5	1.36	0.55, 3.40	22.1
Region						
Northeast	1.00		52.4	1.00		34.7
Midwest	1.10	0.53, 2.27	54.5	0.68	0.29, 1.57	27.4
South	0.75	0.38, 1.46	46.0	0.45	0.19, 1.07	20.6
West	1.01	0.52, 1.98	52.7	0.18 **	0.06, 0.56	9.9
Student race/ethnicity						
Predominantly (≥66%) White	1.00		58.9	1.00		15.8
Majority (≥50%) Black	0.78	0.31, 1.99	53.4	3.04	0.97, 9.56	32.1
Majority (≥50%) Latino	0.41 *	0.18, 0.95	39.4	2.95	0.90, 9.72	31.6
Other/diverse	0.64	0.36, 1.16	49.2	1.51	0.72, 3.16	21.0
School administrative support						
Never/Rarely	1.00		33.1	1.00		4.9
Sometimes	1.56	0.54, 4.49	42.8	3.47	0.80, 15.13	14.5
Often	3.26	1.16, 9.13	59.6	8.55 **	2.05, 35.61	27.9
Financial support						
No/Don’t know	1.00		43.8	1.00		15.9
Yes	1.89 **	1.19, 2.99	58.1	2.13 **	1.25, 3.62	27.0
School wellness champion						
No/Don’t know	1.00		47.0	1.00		21.8
Yes	1.32	0.85, 2.03	53.0	0.94	0.53, 1.68	21.0
Number of schools in model	465			425		

^1^ Note: FRPL = free/reduced-priced lunch (a proxy for financial disadvantage). * *p* < 0.05, ** *p* < 0.01.

## Data Availability

To request access to deidentified data, please contact the corresponding author.

## References

[1] U.S. Department of Health and Human Services (2018). 2018 Physical Activity Guidelines Advisory Committee Scientific Report.

[2] Guthold R., Stevens G.A., Riley L.M., Bull F.C. (2019). Global Trends in Insufficient Physical Activity among Adolescents: A Pooled Analysis of 298 Population-Based Surveys with 16 Million Participants. Lancet Child Adolesc. Health.

[3] Institute of Medicine (2013). Educating the Student Body: Taking Physical Activity and Physical Education to School.

[4] Centers for Disease Control and Prevention (2019). Increasing Physical Education and Physical Activity: A Framework for Schools 2019.

[5] Webster C.A., Russ L., Vazou S., Goh T.L., Erwin H. (2015). Integrating Movement in Academic Classrooms: Understanding, Applying and Advancing the Knowledge Base. Obes. Rev..

[6] Centers for Disease Control and Prevention (2018). Strategies for Classroom Physical Activity in Schools.

[7] Riley N., Lubans D.R., Holmes K., Morgan P.J. (2016). Findings from the EASY Minds Cluster Randomized Controlled Trial: Evaluation of a Physical Activity Integration Program for Mathematics in Primary Schools. J. Phys. Act. Health.

[8] Physical Activity Guidelines for Americans Midcourse Report Subcommittee of the President’s Council on Fitness Sports and Nutrition (2012). Physical Activity Guidelines for Americans Midcourse Report: Strategies to Increase Physical Activity among Youth.

[9] Masini A., Marini S., Gori D., Leoni E., Rochira A., Dallolio L. (2019). Evaluation of School-Based Interventions of Active Breaks in Primary Schools: A Systematic Review and Meta-Analysis. J. Sci. Med. Sport.

[10] Norris E., Steen T.V., Direito A., Stamatakis E. (2019). Physically Active Lessons in Schools and Their Impact on Physical Activity, Educational, Health and Cognition Outcomes: A Systematic Review and Meta-Analysis. Br. J. Sports Med..

[11] Daly-Smith A.J., Zwolinsky S., McKenna J., Tomporowski P.D., Defeyter M.A., Manley A. (2018). Systematic Review of Acute Physically Active Learning and Classroom Movement Breaks on Children’s Physical Activity, Cognition, Academic Performance and Classroom Behaviour: Understanding Critical Design Features. BMJ Open Sport Exerc. Med..

[12] National Physical Activity Plan Alliance (2018). The 2018 United States Report Card on Physical Activity for Children and Youth.

[13] Active Healthy Kids Australia (2018). Muscular Fitness: It’s Time for a Jump Start. The 2018 Active Healthy Kids Australia Report Card on Physical Activity for Children and Young People.

[14] Wilkie H., Standage M., Sherar L., Cumming S., Parnell C., Davis A., Foster C., Jago R. (2016). Results From England’s 2016 Report Card on Physical Activity for Children and Youth. J. Phys. Act. Health.

[15] Aira A., Kämppi K. (2017). Towards More Active and Pleasant School Days. Interim Report on the Finnish Schools on the Move Programme.

[16] Maddison R., Marsh S., Hinckson E., Duncan S., Mandic S., Taylor R., Smith M. (2016). Results From New Zealand’s 2016 Report Card on Physical Activity for Children and Youth. J. Phys. Act. Health.

[17] U.S. Department of Health and Human Services & Centers for Disease Control and Prevention (2014). Results from the School Health Policies and Practices Study 2014.

[18] Turner L., Chaloupka F.J. (2017). Reach and Implementation of Physical Activity Breaks and Active Lessons in Elementary School Classrooms. Health Educ. Behav..

[19] Proctor E., Silmere H., Raghavan R., Hovmand P., Aarons G., Bunger A., Griffey R., Hensley M. (2010). Outcomes for Implementation Research: Conceptual Distinctions, Measurement Challenges, and Research Agenda. Adm. Policy Ment. Health Ment. Health Serv. Res..

[20] Cradock A.L., Barrett J.L., Carter J., McHugh A., Sproul J., Russo E.T., Dao-Tran P., Gortmaker S.L. (2014). Impact of the Boston Active School Day Policy to Promote Physical Activity among Children. Am. J. Health Promot..

[21] Delk J., Springer A.E., Kelder S.H., Grayless M. (2014). Promoting Teacher Adoption of Physical Activity Breaks in the Classroom: Findings of the Central Texas CATCH Middle School Project. J. Sch. Health.

[22] Nathan N.K., Sutherland R.L., Hope K., McCarthy N.J., Pettett M., Elton B., Jackson R., Trost S.G., Lecathelinais C., Reilly K. (2020). Implementation of a School Physical Activity Policy Improves Student Physical Activity Levels: Outcomes of a Cluster-Randomized Controlled Trial. J. Phys. Act. Health.

[23] Naylor P.J., Macdonald H.M., Reed K.E., McKay H.A. (2006). Action Schools! BC: A Socioecological Approach to Modifying Chronic Disease Risk Factors in Elementary School Children. Prev. Chronic. Dis..

[24] Reed K.E., Warburton D.E.R., Macdonald H.M., Naylor P.J., McKay H.A. (2008). Action Schools! BC: A School-Based Physical Activity Intervention Designed to Decrease Cardiovascular Disease Risk Factors in Children. Prev. Med..

[25] Naylor P.-J., Macdonald H.M., Zebedee J.A., Reed K.E., McKay H.A. (2006). Lessons Learned from Action Schools! BC—An ‘Active School’ Model to Promote Physical Activity in Elementary Schools. J. Sci. Med. Sport.

[26] Michael R.D., Webster C.A., Egan C.A., Nilges L., Brian A., Johnson R., Carson R.L. (2019). Facilitators and Barriers to Movement Integration in Elementary Classrooms: A Systematic Review. Res. Q. Exerc. Sport.

[27] Nathan N., Elton B., Babic M., McCarthy N., Sutherland R., Presseau J., Seward K., Hodder R., Booth D., Yoong S.L. (2018). Barriers and Facilitators to the Implementation of Physical Activity Policies in Schools: A Systematic Review. Prev. Med..

[28] Brown K.M., Elliott S.J. (2015). “It’s Not as Easy as Just Saying 20 Minutes a Day”: Exploring Teacher and Principal Experiences Implementing a Provincial Physical Activity Policy. Univers. J. Public Health.

[29] Evenson K.R., Ballard K., Lee G., Ammerman A. (2009). Implementation of a School-Based State Policy to Increase Physical Activity. J. Sch. Health.

[30] Goh T.L., Hannon J.C., Newton M., Webster C., Podlog L., Pillow W. (2013). “I’ll Squeeze It In”: Transforming Preservice Classroom Teachers’ Perceptions Toward Movement Integration in Schools. Action Teach. Educ..

[31] Webster C.A., Zarrett N., Cook B.S., Egan C., Nesbitt D., Weaver R.G. (2017). Movement Integration in Elementary Classrooms: Teacher Perceptions and Implications for Program Planning. Eval. Program Plan..

[32] Carlson J.A., Engelberg J.K., Cain K.L., Conway T.L., Geremia C., Bonilla E., Kerner J., Sallis J.F. (2017). Contextual Factors Related to Implementation of Classroom Physical Activity Breaks. Transl. Behav. Med..

[33] Turner L., Calvert H.G., Carlson J.A. (2019). Supporting Teachers’ Implementation of Classroom-Based Physical Activity. Transl. J. ACSM.

[34] Lau E., Wandersman A., Pate R. (2016). Factors Influencing Implementation of Youth Physical Activity Interventions: An Expert Perspective. Transl. J. Am. Coll. Sports Med..

[35] Carson R.L., Castelli D.M., Pulling Kuhn A.C., Moore J.B., Beets M.W., Beighle A., Aija R., Calvert H.G., Glowacki E.M. (2014). Impact of Trained Champions of Comprehensive School Physical Activity Programs on School Physical Activity Offerings, Youth Physical Activity and Sedentary Behaviors. Prev. Med..

[36] Webster C.A., Beets M., Weaver R.G., Vazou S., Russ L. (2015). Rethinking Recommendations for Implementing Comprehensive School Physical Activity Programs: A Partnership Model. Quest.

[37] Moore J.B., Carson R.L., Webster C.A., Singletary C.R., Castelli D.M., Pate R.R., Beets M.W., Beighle A. (2018). The Application of an Implementation Science Framework to Comprehensive School Physical Activity Programs: Be a Champion!. Front. Public Health.

[38] Heidorn B., Centeio E. (2012). The Director of Physical Activity and Staff Involvement. J. Phys. Educ. Recreat. Dance.

[39] Turner L., Sandoval A., Chaloupka F.J. (2015). Bridging the Gap’s Food and Fitness Elementary School Survey: Technical Report on Survey Development, Sampling, and Methodology.

[40] National Center for Education Statistics Common Core of Data. https://nces.ed.gov/ccd/.

[41] Damschroder L.J., Aron D.C., Keith R.E., Kirsh S.R., Alexander J.A., Lowery J.C. (2009). Fostering Implementation of Health Services Research Findings into Practice: A Consolidated Framework for Advancing Implementation Science. Implement. Sci..

[42] Maidique M.A. (1980). Entrepeneurs, Champions and Technological Innovation. Sloan Manag. Rev..

[43] Snyder T., Musu-Gillette L. Free or Reduced Price Lunch: A Proxy for Poverty?. https://nces.ed.gov/blogs/nces/post/free-or-reduced-price-lunch-a-proxy-for-poverty.

[44] Stata Survey Data Reference Manual, Release 16. https://www.stata.com/bookstore/survey-data-reference-manual/.

[45] Howie E.K., Beets M.W., Pate R.R. (2014). Acute Classroom Exercise Breaks Improve On-Task Behavior in 4th and 5th Grade Students: A Dose–Response. Ment. Health Phys. Act..

[46] Vazou S., Webster C.A., Stewart G., Candal P., Egan C.A., Pennell A., Russ L.B. (2020). A Systematic Review and Qualitative Synthesis Resulting in a Typology of Elementary Classroom Movement Integration Interventions. Sports Med. Open.

[47] Watson A., Timperio A., Brown H., Best K., Hesketh K.D. (2017). Effect of Classroom-Based Physical Activity Interventions on Academic and Physical Activity Outcomes: A Systematic Review and Meta-Analysis. Int. J. Behav. Nutr. Phys. Act..

[48] Allison K.R., Vu-Nguyen K., Ng B., Schoueri-Mychasiw N., Dwyer J.J.M., Manson H., Hobin E., Manske S., Robertson J. (2016). Evaluation of Daily Physical Activity (DPA) Policy Implementation in Ontario: Surveys of Elementary School Administrators and Teachers. BMC Public Health.

[49] Mavilidi M.F., Vazou S. (2021). Classroom-based Physical Activity and Math Performance: Integrated Physical Activity or Not?. Acta Paediatr..

[50] Hasson R., Eisman A., Watkins D., Beemer L., Ajibewa T. Utilizing Implementation Strategies to Increase Equity in Classroom-Based Physical Activity Programming among Low-Resource Schools. Proceedings of the 12th Annual Conference on the Science of Dissemination and Implementation in Health.

[51] Mahar M.T., Kenny R.K., Shields A.T., Scales D.P., Collins B.S. (2006). Energizers Classroom-Based Physical Activities 3–5: The Way Teachers Integrate Physical Activity with Academic Concepts.

[52] Singletary C.R., Weaver G., Carson R.L., Beets M.W., Pate R.R., Saunders R.P., Peluso A.G., Moore J.B. (2019). Evaluation of a Comprehensive School Physical Activity Program: Be a Champion!. Eval. Program Plann..

[53] Lane H.G., Deitch R., Wang Y., Black M.M., Dunton G.F., Aldoory L., Turner L., Parker E.A., Henley S.C., Saksvig B. (2018). “Wellness Champions for Change,” a Multi-Level Intervention to Improve School-Level Implementation of Local Wellness Policies: Study Protocol for a Cluster Randomized Trial. Contemp. Clin. Trials.

[54] Webster C.A., Starrett A., Rehling J., Chen B., Beets M.W., Weaver R.G. (2020). Understanding Elementary Classroom Teachers’ Use of Movement Integration Resources. Front. Educ..

[55] Holt E., Bartee T., Heelan K. (2013). Evaluation of a Policy to Integrate Physical Activity Into the School Day. J. Phys. Act. Health.

[56] Michael S.L., Merlo C.L., Basch C.E., Wentzel K.R., Wechsler H. (2015). Critical Connections: Health and Academics. J. Sch. Health.

